# Impact of nutrition and physical activity on outcomes of hospital-acquired pneumonia

**DOI:** 10.1038/s41598-022-19793-2

**Published:** 2022-09-16

**Authors:** Jin Ho Jang, Taehwa Kim, Hye Ju Yeo, Woo Hyun Cho, Kyung Hoon Min, Jee Youn Oh, Sang-Bum Hong, Ae-Rin Baek, Hyun-Kyung Lee, Changhwan Kim, Youjin Chang, Hye Kyeong Park, Heung Bum Lee, Soohyun Bae, Jae Young Moon, Kwang Ha Yoo, Hyun-Il Gil, Beomsu Shin, Kyeongman Jeon, the Korean HAP/VAP Study Group, Woo Hyun Cho, Kyung Hoon Min, Jee Youn Oh, Sang-Bum Hong, Ae-Rin Baek, Hyun-Kyung Lee, Changhwan Kim, Youjin Chang, Hye Kyeong Park, Heung Bum Lee, Soohyun Bae, Jae Young Moon, Kwang Ha Yoo, Hyun-Il Gil, Beomsu Shin, Kyeongman Jeon

**Affiliations:** 1 Division of Pulmonology, Allergy and Critical Care Medicine, Department of Internal Medicine, Research Institute for Convergence of Biomedical Science and Technology, Pusan National University Yangsan Hospital, Geumo-ro 20, Beomeo-ri, Mulgeum-eup, Yangsan-si, Gyeongsangnam-do 626-770 Republic of Korea; 2 Department of Internal Medicine, School of Medicine, Pusan National University, 49, Busandaehak-ro, Mulgeum-eup, Yangsan-si, Gyeongsangnam-do 626-870 Republic of Korea; 3 Division of Pulmonary, Allergy, and Critical Care Medicine, Department of Internal Medicine, Korea University Guro Hospital, Seoul, Republic of Korea; 4 Department of Pulmonary and Critical Care Medicine, Asan Medical Center, University of Ulsan College of Medicine, Seoul, Republic of Korea; 5 Division of Allergy and Respiratory Medicine, Department of Internal Medicine, Soon Chun Hyang University Bucheon Hospital, Bucheon, Republic of Korea; 6 Department of Internal Medicine, Division of Pulmonology, Allergy and Critical Care Medicine, Busan Paik Hospital, Inje University College of Medicine, Busan, Republic of Korea; 7 Department of Internal Medicine, Jeju National University Hospital, Jeju National University School of Medicine, Jeju, Republic of Korea; 8 Division of Pulmonary and Critical Care Medicine, Department of Internal Medicine, Inje University Sanggye Paik Hospital, Seoul, Republic of Korea; 9 Division of Pulmonary and Critical Care Medicine, Department of Internal Medicine, Ilsan Paik Hospital, Inje University College of Medicine, Ilsan, Republic of Korea; 10 Department of Internal Medicine, Research Center for Pulmonary Disorders, Jeonbuk National University Medical School and Hospital, Jeonju, Republic of Korea; 11 Department of Integrated Internal Medicine, Myoungji Hospital, Hanyang University Medical Center, Goyang, Republic of Korea; 12 Division of Pulmonary and Critical Care Medicine, Department of Internal Medicine, Chungnam National University Sejong Hospital, Sejong, Republic of Korea; 13 Division of Pulmonary, Allergy, and Critical Care Medicine, Department of Internal Medicine, Konkuk University School of Medicine, Seoul, Republic of Korea; 14 Division of Pulmonary and Critical Care Medicine, Department of Internal Medicine, Kangbuk Samsung Hospital, Sungkyunkwan University School of Medicine, Seoul, Republic of Korea; 15 Division of Pulmonary and Critical Care Medicine, Department of Medicine, Samsung Changwon Hospital, Sungkyunkwan University School of Medicine, Changwon, Republic of Korea; 16 Division of Pulmonary and Critical Care Medicine, Department of Medicine, Samsung Medical Center, Sungkyunkwan University School of Medicine, Seoul, Republic of Korea

**Keywords:** Respiratory tract diseases, Medical research, Risk factors

## Abstract

Frailty is an important risk factor for adverse health-related outcomes. It is classified into several phenotypes according to nutritional state and physical activity. In this context, we investigated whether frailty phenotypes were related to clinical outcome of hospital-acquired pneumonia (HAP). During the study period, a total of 526 patients were screened for HAP and 480 of whom were analyzed. The patients were divided into four groups according to physical inactivity and malnutrition: nutritional frailty (Geriatric Nutritional Risk Index [GNRI] < 82 and Clinical Frailty Scale [CFS] ≥ 4), malnutrition (GNRI < 82 and CFS < 4), physical frailty (GNRI ≥ 82 and CFS ≥ 4), and normal (GNRI ≥ 82 and CFS < 4). Among the phenotypes, physical frailty without malnutrition was the most common (39.4%), followed by nutritional frailty (30.2%), normal (20.6%), and malnutrition (9.8%). There was a significant difference in hospital survival and home discharge among the four phenotypes (p = 0.009), and the nutritional frailty group had the poorest in-hospital survival and home discharge (64.8% and 34.6%, respectively). In conclusion, there were differences in clinical outcomes according to the four phenotypes of HAP. Assessment of frailty phenotypes during hospitalization may improve outcomes through adequate nutrition and rehabilitation treatment of patients with HAP.

## Introduction

Frailty is an important risk factor for adverse health-related outcomes including falls, hospitalization, dementia, and mortality. It is characterized by increased vulnerability to poor resolution of homeostasis after a stressor and is associated with increased severity and mortality in infectious diseases^1–3^. This condition affects multiple domains of human functioning and has a multidimensional nature based on different pathophysiological mechanisms. There are several phenotypes, such as nutritional frailty, sarcopenia, and physical frailty, according to nutritional state and physical activity^4^. Both low physical activity and malnutrition play a significant role in the onset and progression of frailty syndrome^5^. They share several risk factors in common, and each factor promotes a vicious cycle by causing weight loss, sarcopenia, and decreased energy expenditure in the frailty cycle^6,7^. Therefore, it is fundamental to identify reversible factors according to the phenotypes to maximize the effect of treatment in frailty.

In a previous studies, we found that each factors including nutritional status, sarcopenia and physical activity was significantly associated with clinical outcomes in various respiratory diseases^8–12^. However, there has been a limitation in that it is not a multidimensional measure. Recently, several studies have shown an association between frailty and mortality in coronavirus disease^13–18^. However, there is a lack of studies considering combined nutritional states.

Hospital-acquired pneumonia (HAP) is one of the most common and morbid hospital-acquired infections^19^. The incidence of HAP is high in patients who are older (elderly patients) or who have comorbidities, longer hospitalization, immune compromise, and high aspiration risk of oropharyngeal material^20^. Therefore, malnutrition and physical inactivity are critical for the development of HAP and the associated outcomes. However, no study has conducted a detailed assessment of frailty phenotypes according to nutritional status and physical activity, and its prognostic significance in HAP. Therefore, we evaluated that each frailty phenotypes, in accordance with physical inactivity or nutritional status, may impact on clinical outcome in HAP.

## Methods

### Study design and population

This study was conducted in the Korean HAP/ventilator-associated pneumonia (VAP) study group. A multicenter retrospective cohort study was conducted at 13 tertiary or university-affiliated hospitals in Korea from July 1, 2019 to December 31, 2019^21^. Regular audits were conducted by an employee of the principal institution (Samsung Medical Center) to verify the quality of the reported data. This study was approved by the institutional review board of each participating hospital, including the Pusan National University Yangsan Hospital Review Board (approval no. 05–2020-067, see Appendix, Supplemental Digital Content S1, which). The need for informed consent from the patients was waived because of the retrospective observational study design (see Appendix, Supplemental Digital Content S2, which). All methods were carried out in accordance with relevant guidelines and regulations. Adult patients aged ≥ 19 years who had a hospitalization period of 3 days or more and those with a pneumonia-related code as per the International Classification of Diseases, 10th revision, (J13-J18, J85) at discharge were screened for eligibility. Patients who developed pneumonia within 48 h after referral from other hospitals or those who received antibiotics for more than 72 h from other hospitals were excluded from this study. HAP was defined according to the 2016 American Thoracic Society / Infectious Disease Society of America guidelines^22^.

### Data collection

Information of the following clinical variables was collected from the case report form: (1) demographic characteristics, including age, sex, body mass index (BMI), Geriatric Nutritional Risk Index (GNRI), Clinical Frailty Scale (CFS), Charlson Comorbidity Index (CCI), and comorbidities; (2) clinical outcomes, including clinical response to HAP, hospital mortality, readmission to the intensive care unit (ICU), and discharge destination.

### Assessment of physical inactivity and malnutrition

We used CFS as an assessment tool to assess the physical inactivity of patients. The CFS was developed from the Canadian Study of Health and Aging and has been widely used to predict the outcomes of older people hospitalized with acute illness^23^. The CFS is a scale from 1 (very fit) to 9 (terminally ill) and evaluates specific domains including comorbidity, function, and cognition^24^. Physical inactivity was defined as a score greater than CFS 4 because level 4 indicated living with limitation of activities^25^. The GNRI was calculated as (1.519 × serum albumin [g/dL] + 41.7 × present weight [kg]/ideal body weight [IBW, kg])^26^. IBW was calculated according to the Lorentz formula, calibrated for the patient’s height and sex. Malnutrition was defined as a GNRI of < 82^27^.

### Definition

Sepsis and septic shock were defined by clinical criteria according to the Third International Consensus Definitions for Sepsis and Septic Shock (Sepsis-3)^28^. Immunocompromised patients were defined as those with CD4 + counts below 200 cells/mm^3^ in human immunodeficiency virus infection, those with neutrophil counts < 1000 cells/mm^3^, or those taking immunosuppressants after organ transplantation. History of using high-dose or long-term steroids was over 20 mg/day of prednisone or its equivalent for at least 2 weeks.

Clinical responses were classified following baseline comparison of symptoms and signs. Clinical cure defined as the improvement of all signs and symptoms associated with pneumonia (e.g. fever, purulent sputum, leukocytosis, and oxygenation)^22^, clinical failure was defined as persistence or worsening of signs, symptoms, or both, associated with pneumonia, or occurring within 3 days after the termination of treatment. Clinical recurrence was defined as occurrence of a new event of pneumonia 72 h after antibiotic discontinuation.

### Statistical analysis

All statistical analyses were performed using MedCalc, version 11.3.6.0 (MedCalc Software, Mariakerke, Belgium), and SPSS, version 26 (IBM Corp., Armonk, NY, USA). Categorical variables are expressed as numbers with percentages, and continuous variables are expressed as means with standard deviations. One-way analysis of variance or Kruskal–Wallis test was used to compare continuous variables, as appropriate. Categorical variables were compared using the χ^2^ test or Fisher’s exact test, as appropriate. Cox regression analysis was performed to identify predictors of hospital mortality and failure of home discharge. We confirmed that all data satisfies the proportional hazard assumption for cox regression analysis using Scheonfeld residuals. The follow-up period for each Cox regression analysis is defined as from admission to discharge of hospital. We selected independent variables based on clinical significance, and the number of events per dependent variable (hospital mortality or failure of home discharge). After univariate analyses, significant (p < 0.05) values were entered into stepwise backward multivariate Cox regression analyses. In the multivariate Cox analysis for hospital mortality, connective tissue disease, immunocompromised state, hematologic malignancies, solid malignant tumors, physical inactivity, SOFA score, sepsis and septic shock were included. In the multivariate Cox analysis for fail to home discharge, solid malignant tumors, physical inactivity, and malnutrition were included. Survival curves and rates were determined by Kaplan–Meier analyses, and group differences in survival were compared using the Breslow test.

## Results

### Baseline characteristics of patients according to frailty phenotypes

**Figure 1:**
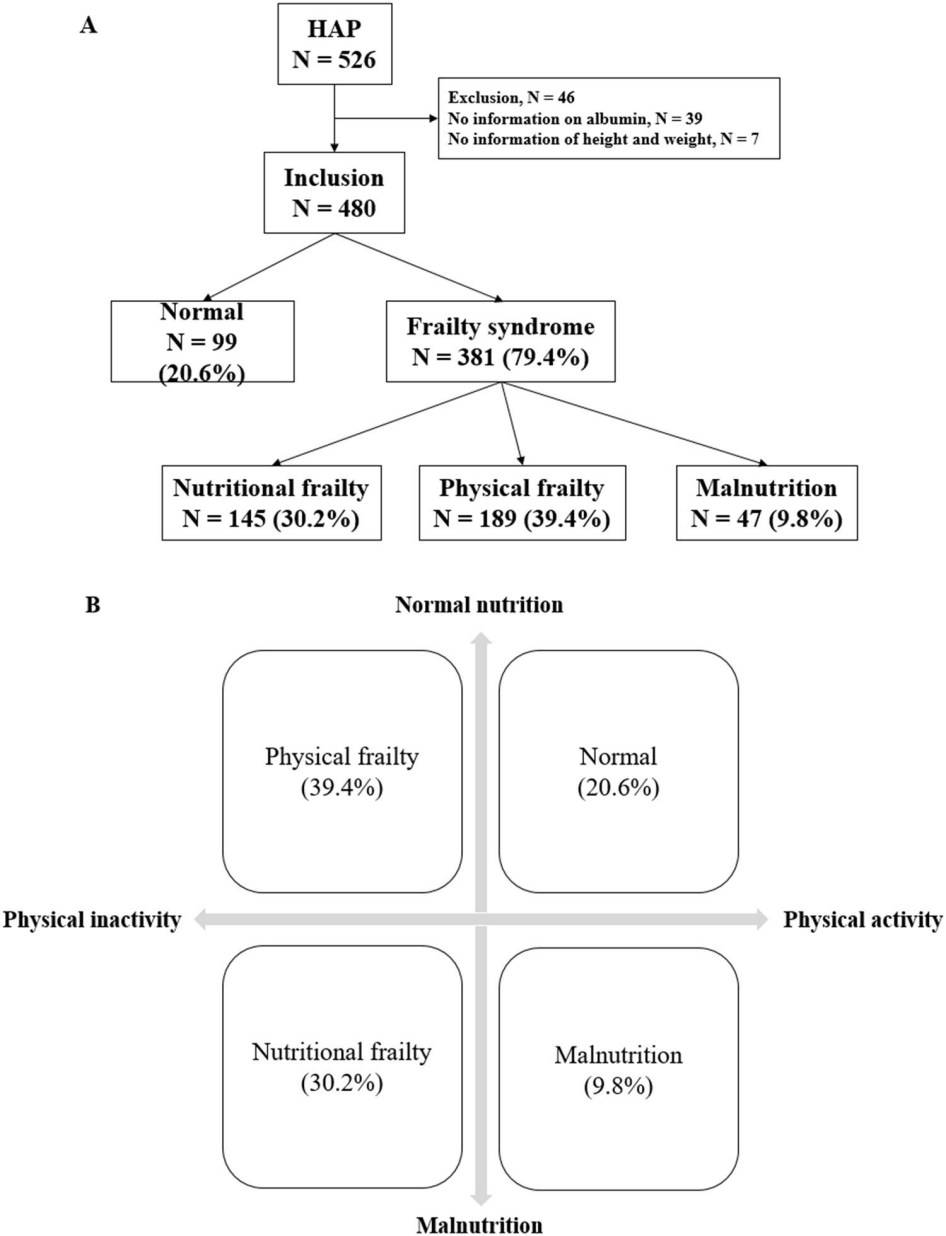
(**A**) Flowchart of the patient enrollment process. During the study period, 526 patients were screened for HAP/VAP. Among them, 480 were included and divided into four groups according to physical inactivity and malnutrition. *HAP* hospital-acquired pneumonia. (**B**) Phenotypes of frailty. Patients were divided into four groups according to physical inactivity and malnutrition: nutritional frailty, physical frailty, malnutrition, and normal.

During the study period, a total of 206,372 patients were hospitalized and 526 of whom were screened for HAP/VAP (2.54/1000 patients). Among them, 480 were analyzed, with the exception of 39 patients who did not undergo measurement of serum albumin and 7 patients who did not have height or weight measurements at the time of admission (Fig. 1A). The included patients were divided into four groups according to physical inactivity and malnutrition (nutritional frailty, physical frailty, malnutrition, and normal group, Fig. 1B). The physical frailty group without malnutrition was the most common (39.4%), followed by nutritional frailty (30.2%), normal (20.6%), and malnutrition (9.8%).

Table 1 shows the baseline characteristics of patients across frailty phenotypes. There was no significant difference in age among the four groups. There was no significant difference in age groups, such as the elderly and very elderly, among the four groups. Mean BMI was significantly lower in the nutritional frailty group than in the other three groups (p < 0.001), and malnutrition, physical frailty, and normal were lower in that order (p < 0.001). In addition, the mean CCI of the nutritional frailty and physical frailty groups was significantly higher than that of the normal group (nutritional frailty vs. normal, p < 0.001; physical frailty vs. normal, p < 0.001). Chronic neurological disease was common in the nutritional frailty and physical frailty groups (34.5% and 32.8%, respectively; p = 0.001). However, comorbidities such as immunocompromised state and solid malignant tumor were most common in the malnutrition group (immunocompromised, p = 0.022; solid malignant tumor, p = 0.019). Sepsis was more frequent in the nutritional frailty, physical frailty, and malnutrition groups than in the normal group (p = 0.007).

**Table 1:** Baseline characteristics according to frailty phenotypes.

Variables	Normal (n = 99)	Nutritional frailty (n = 145)	Physical frailty (n = 189)	Malnutrition (n = 47)	*p* value
Age	68.3 ± 13.3	70.2 ± 12.3	70.1 ± 13.1	67.4 ± 14.1	0.395
**Age group**					
Non-elderly < 65	31 (31.3%)	45 (31.0%)	63 (33.3%)	17 (36.2%)	0.166
Elderly 65–74	37 (37.4%)	40 (27.6%)	42 (22.2%)	14 (29.8%)	
Very elderly 75-	31 (31.3%)	60 (41.4%)	84 (44.4%)	16 (34.0%)	
Male	66 (66.7%)	103 (71.0%)	128 (67.7%)	31 (66.0%)	0.856
BMI	24.8 ± 4.1	19.1 ± 2.7	23.6 ± 3.6	19.8 ± 3.1	< 0.001
GNRI	94.3 ± 8.5	73.0 ± 5.3	94.3 ± 8.5	74.2 ± 5.3	< 0.001
CFS	2.4 ± 0.7	6.4 ± 1.6	6.1 ± 1.6	2.6 ± 0.7	< 0.001
CCI	3.8 ± 2.1	5.0 ± 2.5	5.2 ± 2.7	4.5 ± 2.6	< 0.001
SOFA score	4.6 ± 4.5	5.7 ± 4.2	5.5 ± 3.9	4.7 ± 3.4	0.118
Cardiovascular disease	19 (19.2%)	27 (18.6%)	47 (24.9%)	7 (14.9%)	0.326
Chronic lung disease	15 (15.2%)	16 (11.0%)	30 (15.9%)	6 (12.8%)	0.619
Chronic neurological disease	13 (13.1%)	50 (34.5%)	62 (32.8%)	10 (21.3%)	0.001
Chronic kidney disease	8 (8.1%)	27 (18.6%)	28 (14.8%)	7 (14.9%)	0.153
Chronic liver disease	6 (6.15)	13 (9.0%)	13 (6.9%)	5 (10.6%)	0.694
Diabetes mellitus	30 (30.3%)	44 (30.3%)	68 (36.0%)	10 (21.3%)	0.244
CTD^†^	2 (2.0%)	4 (2.8%)	8 (4.2%)	2 (4.3%)	0.735
Immunocompromised^a^	2 (2.0%)	8 (5.5%)	11 (5.8%)	7 (14.9%)	0.022
Hematological malignancies	3 (3.0%)	10 (6.9%)	19 (10.1%)	4 (8.5%)	0.189
Solid malignant tumors	38 (38.4%)	52 (35.9%)	53 (28.0%)	24 (51.1%)	0.019
High dose or long-term corticosteroid use^b^	2 (2.0%)	14 (9.7%)	12 (6.3%)	5 (10.6%)	0.089
Prior IV antibiotics use within 90 days	72 (72.7%)	105 (72.4%)	133 (70.4%)	38 (80.9%)	0.557
Artificial airway^c^	25 (25.3%)	50 (34.55)	57 (30.25)	12 (25.5%)	0.407
Risk of aspiration^d^	60 (60.6%)	97 (66.9%)	124 (65.6%)	27 (57.4%)	0.550
Sepsis	51 (51.5%)	105 (72.4%)	125 (66.1%)	33 (70.2%)	0.007
Septic shock	12 (12.1%)	16 (11.0%)	21 (11.1%)	5 (10.6%)	0.991

Data are presented as mean ± standard deviation or as number (%).

*BMI* body mass index, *GNRI* geriatric nutritional risk index, *CFS* Clinical Frailty Scale, *CCI* Charlson Comorbidity Index, *SOFA* sequential organ failure assessment, *CTD* connective tissue disease, *IV* intravenous.

^a^HIV-infected patients with CD4 + counts of less than 200 cells/mm^3^, neutrophil counts of less than 1000 cells/mm^3^, or patients taking immunosuppressive drugs after organ transplantation.

^b^Prednisone 20 mg/day or its equivalent taken for at least 2 weeks.

^c^Tracheostomy tube, tracheal tube, and others.

^d^Impaired swallowing (esophageal disease, neurologic disease, and recent extubation), impaired consciousness, increased chances of gastric contents reaching the lung (reflux and tubal feeding), and impaired cough reflex (medications, stroke, and dementia).

^†^Fisher’s exact test was conducted.

### Clinical outcomes according to frailty phenotypes

Clinical outcomes are shown in Table 2. The clinical cure rate was higher in the normal group than in the other groups (p = 0.006). There were no significant differences in the length of hospital stay, ICU readmission, and step-down or step-up referral. Hospital survival showed a significant difference among the four groups, and the survival rate of the nutritional frailty group was the lowest (64.8%, p = 0.009). In addition, home discharge had a significant difference among the four groups, and the proportion of home discharge was lowest in the nutritional frailty group (43.6%, p = 0.040).

**Table 2:** Clinical outcomes according to phenotypes of frailty.

Variables	Normal (n = 99)	Nutritional frailty (n = 145)	Physical frailty (n = 189)	Malnutrition (n = 47)	*p* value
**Clinical response**^**†**^					
Clinical cure^a^	80 (80.8%)	84 (57.9%)	113 (59.8%)	33 (70.2%)	0.006
Clinical failure^b^	17 (17.2%)	59 (40.7%)	71 (37.6%)	13 (27.7%)	
Clinical recurrence^c^	2 (2.0%)	2 (1.4%)	5 (2.6%)	1 (2.1%)	
Hospital stay, days^††^	25 (14–45)	31 (19.5–62.0)	31 (18.5–52)	35 (24–63)	0.054
Hospital survival	83 (83.8%)	94 (64.8%)	131 (69.3%)	36 (76.6%)	0.009
ICU readmission	13 (13.1%)	41 (28.3%)	55 (29.1%)	17 (36.2%)	0.006
Home discharge	54 (65.1%)	41 (43.6%)	68 (51.9%)	20 (55.6%)	0.040
**Transfer**^**†**^					
Step down referral	25 (25.3%)	47 (32.4%)	61 (32.3%)	15 (31.9%)	0.247
Step up referral	4 (4.0%)	6 (4.1%)	2 (1.1%)	1 (2.1%)	

Data are presented as mean ± standard deviation, median (IQR), or number (%).

^†^Fisher’s exact test was conducted.

^††^Data are presented as median [interquartile range]. Kruskal–Wallis test was used.

*ICU* intensive care unit, *IQR* interquartile range.

^a^Improvement of all signs and symptoms associated with pneumonia.

^b^Persistence or worsening of signs, symptoms, or both, associated with pneumonia, symptoms, signs of pneumonia, or both, occurring again within 3 days after termination of treatment.

^c^Occurrence of a new event of pneumonia 72 h after antibiotic discontinuation.

### Predictors of hospital mortality for patients with HAP

The results of the Cox proportional hazards regression are presented in Table 3. Connective tissue disease (CTD), immunocompromised state, hematological malignancies, solid malignant tumors, physical inactivity (CFS ≥ 4), initial Sequential Organ Failure Assessment (SOFA) score, sepsis, and septic shock were significantly associated with hospital mortality in the univariate analysis, but CTD (hazard ratio [HR] 2.50; 95% confidence interval [CI] 1.12–5.54; p = 0.025), hematological malignancies (HR, 2.45; 95% CI 1.50–4.02; p < 0.001), solid malignant tumors (HR, 1.72; 95% CI 1.20–2.48; p = 0.003), physical inactivity (HR, 1.64; 95% CI 1.07–2.51; p = 0.023), and initial SOFA score (HR, 1.08; 95% CI 1.03–1.13; p = 0.001) remained significant in the multivariate analysis.

**Table 3:** Results of Cox regression analysis for hospital mortality.

Variables	Univariate		Multivariate	
	HR (95% CI)	P	HR (95% CI)	P
Connective tissue disease	2.29 (1.06–4.92)	0.034	2.50 (1.12–5.54)	0.025
Immunocompromised state^a^	1.95 (1.16–3.30)	0.012		
Hematological malignancies	1.78 (1.12–2.84)	0.015	2.45 (1.50–4.02)	< 0.001
Solid malignant tumors	1.42 (1.01–2.00)	0.043	1.72 (1.20–2.48)	0.003
Physical inactivity (CFS ≥ 4)	1.64 (1.08–2.50)	0.021	1.64 (1.07–2.51)	0.023
SOFA score	1.10 (1.06–1.14)	< 0.001	1.08 (1.03–1.13)	0.001
Sepsis	1.56 (1.05–2.29)	0.026		
Septic shock	2.31 (1.55–3.45)	< 0.001		

*HR* hazard ratio, *CI* confidence interval, *CFS* clinical frailty scale, *SOFA* sequential organ failure assessment.

^a^HIV-infected patients with CD4 + counts of less than 200 cells/mm^3^, neutrophil counts of less than 1000 cells/mm^3^, or patients taking immunosuppressive drugs after organ transplantation.

**Figure 2:**
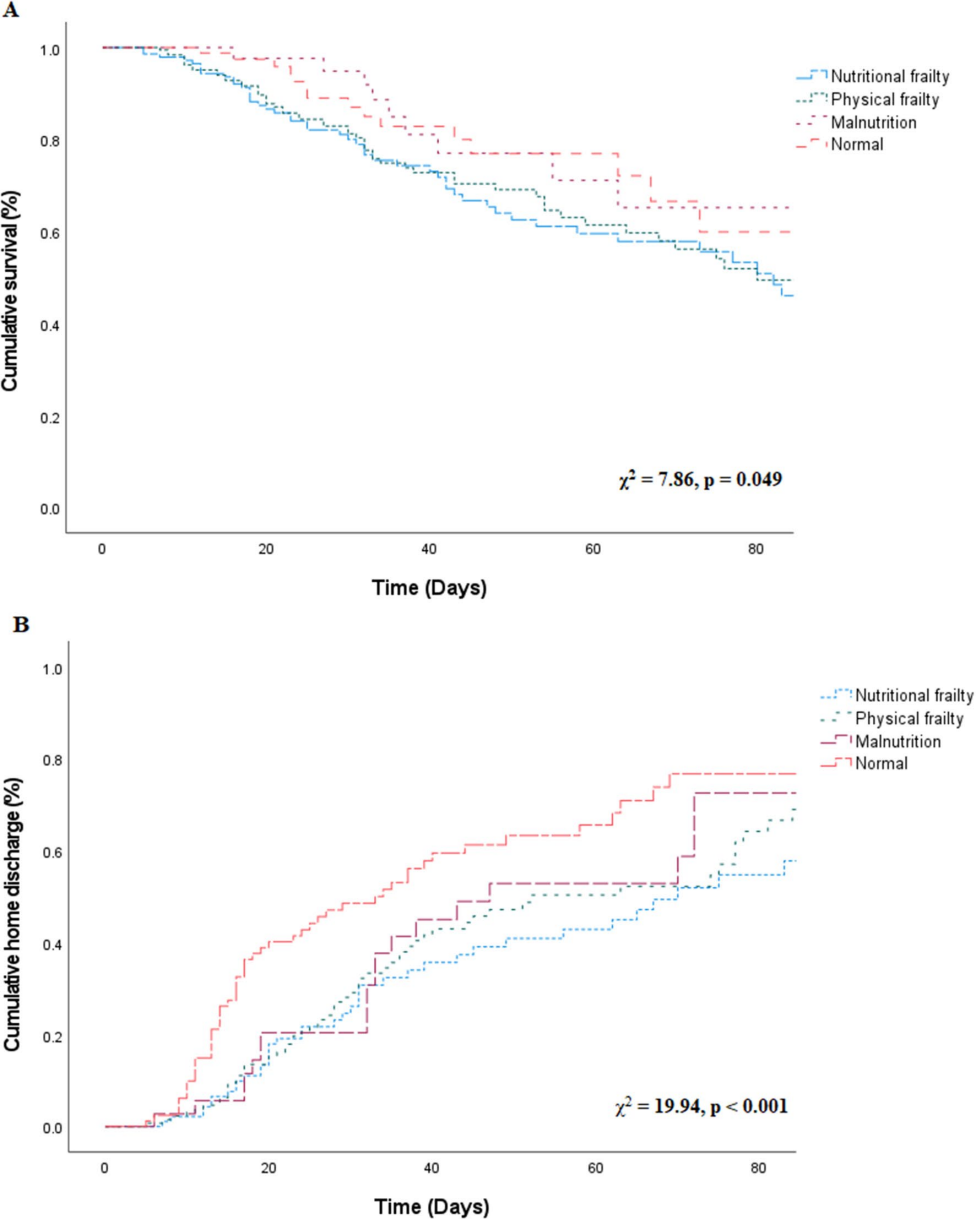
(**A**) Survival according to phenotypes of frailty. There was a significant difference in the cumulative survival possibility among the four phenotypes. (**B**) Home discharge according to phenotypes of frailty. There was a significant difference in the cumulative home discharge possibility among the four phenotypes.

The Kaplan–Meier survival curve showed that nutritional frailty resulted in a higher risk of mortality (χ^2^ = 7.86, p = 0.049, Fig. 2A). The risk of hospital-related mortality was high in the order of the nutritional frailty, physical frailty, malnutrition, and normal groups (p = 0.009).

### Predictors of failure to home discharge

Cox proportional hazards regression analysis was performed to verify the predictors of failure to home discharge (Table 4). In the univariate Cox analysis, solid malignant tumor (HR, 0.65; 95% CI 0.49–0.88; p = 0.005), physical inactivity (HR, 1.49; 95% CI 1.11–2.00; p = 0.009), and malnutrition (HR, 1.44; 95% CI 1.06–1.96; p = 0.021) were significant factors. In the multivariate Cox analysis, solid malignant tumor (HR, 0.65; 95% CI 0.48–0.88; p = 0.005), physical inactivity (HR, 1.43; 95% CI, 1.07 to 1.93; p = 0.017), and malnutrition (HR, 1.43; 95% CI 1.05–1.94; p = 0.024) continued to be significantly associated with failure to home discharge.

**Table 4:** Cox regression for fail to home discharge.

Variables	Univariate		Multivariate	
	OR (95% CI)	P	OR (95% CI)	P
Solid malignant tumors	0.65 (0.49–0.88)	0.005	0.65 (0.48–0.88)	0.005
Physical inactivity (CFS ≥ 4)	1.49 (1.11–2.00)	0.009	1.43 (1.07–1.93)	0.017
Malnutrition (GNRI < 82)	1.44 (1.06–1.96)	0.021	1.43 (1.05–1.94)	0.024

*OR* odds ratio, *CI* confidence interval, *CFS* Clinical Frailty Scale, *GNRI* Geriatric Nutritional Risk Index.

As shown in Fig. 2B, nutritional frailty caused a higher risk of failure to home discharge (χ^2^ = 19.94, p < 0.001). The probability of being discharged to home was highest in the normal, followed by malnutrition, physical frailty, and nutritional frailty groups (p = 0.040).

## Discussion

In this study, the clinical characteristics and outcomes of HAP were significantly different in each frailty phenotype according to physical activity and nutritional status. A total of 80% of patients have been accompanied by physical inactivity and/or malnutrition. Among the phenotypes, the physical frailty group without malnutrition was most common (39.4%), followed by nutritional frailty, both malnutrition and physical inactivity, and was the second most common (30.2%). Survival and function status at hospital discharge were the worst in the nutritional frailty group. Physical inactivity and malnutrition were significantly associated with hospital-related mortality and functional status at discharge. Therefore, both physical inactivity and malnutrition are critical for complete functional recovery and survival in patients with HAP.

Recently, frailty syndrome has been considered important in the prognosis of pneumonia, but there is still a lack of understanding of this condition in HAP. Frailty syndrome is a complex concept with a multidimensional perspective and reversible clinical conditions. Despite the widespread importance of this syndrome in clinical management, there is no consensus or guideline regarding specific phenotypes. Classification of phenotypes is essential to clinically predict the response to treatment and to individualize therapeutic approaches such as nutrition or rehabilitation^29,30^. Early screening of each phenotype facilitates a multidisciplinary team-based approach for patients with priority, and this may consequently improve prognosis.

In this study, most patients (80%) had either physical inactivity or malnutrition, and approximately one-third had both. Regardless of age, these two factors are well-documented problems in hospitalized populations^31,32^. Baseline physical inactivity and malnutrition were significantly associated with medically or physically poor conditions at the time of discharge in HAP. Both factors may result in the loss of muscle strength and function and physical dysfunction, as well as increased inflammation and oxidative stress^5,8^. These effects may be more prominent in sarcopenia patients or malnourished patients who are relatively more vulnerable. Unfortunately, this study could not clearly reveal the muscle status of patients. Therefore, further research is required to clarify this issue.

This study had limitations, which should be considered. First, the study had a retrospective design. Among the many problems created by this design, one of which is about the accuracy of diagnosis of pneumonia. It seems likely that, several diagnosis of pneumonia may have been missed, because the patients were enrolled in the study using the hospital code of pneumonia. In fact, the prevalence of HAP in our study was smaller than in other studies^33^. Second, the registry data recorded only hospital-related mortality. Therefore, the results may not reflect the full scope of long-term mortality associated with frailty. Third, physical inactivity and malnutrition were defined using the CFS and GNRI, respectively, which are static measurements. They did not include longitudinal measurements of physical impairment or nutritional status. Despite these limitations, this study included a large-scale population of patients with HAP as a multicenter registry. Given the limited sample size and design, it is not enough to draw a causality from this study. However, this data is raising possible question for future analysis. GNRI and CFS are representative objective scoring systems that well reflect malnutrition and physical inactivity, respectively^24,34,35^. To date, no study has investigated the phenotypes of frailty and its prognostic significance in HAP. This study provides concrete insight into the importance of physical inactivity and malnutrition in HAP. Early recognition and correction of physical inactivity and malnutrition may not only increase survival but also reduce the physical sequelae. Further studies on specific therapeutic approaches depending on the frailty phenotypes in HAP are required.

## Conclusion

Based on physical activity and nutritional status, frailty syndrome, which affects the prognosis of HAP, was divided into four phenotypes. Physical inactivity and malnutrition negatively affected hospital outcomes in HAP patients. Physical and nutritional phenotyping of frailty suggests a novel prognostic perspective and therapeutic target in this population. Assessment of physical inactivity and malnutrition is required during hospitalization for adequate nutritional support and rehabilitation treatment. Further research is warranted to clarify the usefulness of the classification of frailty syndrome phenotypes for prognostication and therapeutic targets.

## Supplementary Information

Supplementary Information.

## Data Availability

The data that support the findings of this study are available from the corresponding authors, W. H. C. and H. J. Y., upon reasonable request.
