# Supplementary material for: Impact of nutrition and physical activity on outcomes of hospital-acquired pneumonia
Source: Sci Rep. 2022 Sep 16;12:15605. doi: 10.1038/s41598-022-19793-2 (PMC9481870; doi:10.1038/s41598-022-19793-2)
Supplement: Supplementary file 1 — Supplementary Information. [file 41598_2022_19793_MOESM1_ESM.docx]

**Supplemental Materials**

**Impact of nutrition and physical activity on outcomes of hospital-acquired pneumonia**

Jin Ho Jang, Taehwa Kim, Hye Ju Yeo, Woo Hyun Cho, Kyung Hoon Min, Sang-Bum Hong, Ae-Rin Baek, Hyun-Kyung Lee, Changhwan Kim, Youjin Chang, Hye Kyeong Park, Jee Youn Oh, Heung Bum Lee, Soohyun Bae, Jae Young Moon, Kwang Ha Yoo, Hyun-Il Gil, Beomsu Shin, Kyeongman Jeon; on behalf of the Korean HAP/VAP Study Group

Table of Contents

| List of Supplemental Digital Content | Page |
| --- | --- |
| Supplemental Digital Content 1. Appendix | 3 |
| Supplemental Digital Content 2. Appendix | 4 |

Supplemental Digital Content 1. Appendix

Institutional review boards of all the participating institutes

| No | Institution |
| --- | --- |
| 1 | Pusan National University Yangsan Hospital |
| 2 | Korea University Guro Hospital |
| 3 | Asan Medical Center |
| 4 | Soon Chun Hyang University Bucheon Hospital |
| 5 | Inje University Busan Paik Hospital |
| 6 | Jeju National University Hospital |
| 7 | Inje University Sanggye Paik Hospital |
| 8 | Inje University Ilsan Paik Hospital |
| 9 | Jeonbuk National University Hospital |
| 10 | Ulsan University Hospital |
| 11 | Chungnam National University Hospital |
| 12 | Konkuk University Hospital |
| 13 | Kangbuk Samsung Hospital |
| 14 | Samsung Changwon Hospital |
| 15 | Samsung Medical Center |

Supplemental Digital Content 2. Appendix

Institutional review boards which waived the need of informed consent from participants

| No | Institution |
| --- | --- |
| 1 | Pusan National University Yangsan Hospital |
| 2 | Korea University Guro Hospital |
| 3 | Asan Medical Center |
| 4 | Soon Chun Hyang University Bucheon Hospital |
| 5 | Inje University Busan Paik Hospital |
| 6 | Jeju National University Hospital |
| 7 | Inje University Sanggye Paik Hospital |
| 8 | Inje University Ilsan Paik Hospital |
| 9 | Jeonbuk National University Hospital |
| 10 | Ulsan University Hospital |
| 11 | Chungnam National University Hospital |
| 12 | Konkuk University Hospital |
| 13 | Kangbuk Samsung Hospital |
| 14 | Samsung Changwon Hospital |
| 15 | Samsung Medical Center |
